# Decreased carbohydrate metabolism enzyme activities in the glaucomatous trabecular meshwork

**Published:** 2010-07-10

**Authors:** Anna K. Junk, Manik Goel, Tom Mundorf, Edward J. Rockwood, Sanjoy K. Bhattacharya

**Affiliations:** 1 Bascom Palmer Eye Institute, University of Miami Miller School of Medicine, Miami, FL; 2 Miami Veterans Affairs Health Care System, Miami, FL; 3 Mundorf Eye Center, Charlotte, NC; 4 Cole Eye Institute, Cleveland Clinic Foundation, Cleveland, OH

## Abstract

**Purpose:**

To determine whether activity of carbohydrate metabolism enzymes (aldolase, pyruvate kinase, isocitrate dehydrogenase, and malate dehydrogenase) are altered in the glaucomatous trabecular meshwork (TM) compared to controls.

**Methods:**

Tissue specimens were obtained from trabeculectomy (n=45 open angle glaucoma; Caucasian, average age 61±8 years of age of both genders) and from cadaver eyes (n=15 control and n=5 glaucoma; Caucasian, average age 63±4 years of both genders). Protein extracts from TM tissue were prepared in a non-denaturing buffer containing 0.1% genapol. Aldolase activity was measured spectrophotometrically at 240 nm absorbance using reaction of 3-phosphoglycerate with hydrazine to form hydrazone. Pyruvate kinase activity was measured by coupling lactate dehydrogenase with NADPH and pyruvate absorbance was measured at 340 nm. Isocitrate dehydrogenase activity was measured using reduction of NADP to NADPH at the characteristic absorbance at 340 nm. Malate dehydrogenase catalyzes the interconversion of L-malate and oxaloacetate using NADP as a coenzyme, quantified by its absorbance at 340 nm.

**Results:**

Aldolase, pyruvate kinase, isocitrate dehydrogenase, and malate dehyrogenase activities in the glaucomatous TM tissue were found to be reduced 70, 50, 25, and 69 percent, respectively. SDS–PAGE analysis suggests the presence of 4-hydorxynonenal (HNE) modified isocitrate dehydrogenase protein in the glaucomatous TM tissue compared to controls.

**Conclusions:**

Several Krebs cycle enzyme activities are considerably reduced in glaucomatous TM. HNE modified isocitrate dehydrogenase activity is consistent with reduced inactivated form of the protein. Lipid peroxidation product modification of aldolase, pyruvate kinase, and isocitrate dehydrogenase serves as a likely reason for the reduction of enzyme activity.

## Introduction

Open angle glaucoma is an irreversible late onset and progressively blinding disease. The anatomy of anterior chamber angle, the site of aqueous egress, between iris and cornea, has been used to differentiate open angle glaucomas from angle closure glaucomas. Primary open angle glaucoma (POAG) is established as a way of exclusion when no inciting condition can be ascribed to development of open angle glaucoma. Glaucomas are termed secondary when they occur in association with prior injury or concomitant illness. Elevated intraocular pressure (IOP) is a risk factor for glaucoma.

Glaucomas have been ascribed to an imbalance in aqueous humor production and outflow. Increased resistance at the level of TM decreases the outflow and leads to elevated IOP [1]. In POAG increased resistance in TM occurs due to changes in the TM cells. Several studies have shown that TM cells and/or their capacity for cell remodeling undergo significant alterations. We reasoned that biochemical changes in the TM have to underlie cellular changes. One of the pathomechanisms expected to be involved is the TM energy metabolism, which affects other processes, such as TM remodeling capacity. We investigated four critical enzymes of energy metabolism (Krebs cycle) in the TM. Previous reports elucidated to the role of protein lipid oxidation modification in the glaucomatous optic nerve [2-4]. We provide evidence that lipid peroxidation product modification reduces the activity of isocitrate dehydrogenase, one of the key enzymes of the Krebs cycle in the glaucomatous TM.

## Methods

### Tissue procurement

Glaucomatous tissue specimens were obtained from trabeculectomy (n=45 open angle glaucoma; Caucasian, age range 53–69 years) and from cadaver eyes (n=5; Caucasian, age range 59–67, encompassing both genders). Cadaver eye derived TM tissue (n=15) from non-glaucomatous donors of both genders (age range 59–67) were used as normal controls. Protein extracts from TM tissue were prepared in a non-denaturing buffer (125 mM Tris-Cl pH 7.0, 100 mM NaCl) containing 0.1% Genapol C-100 (a detergent used to extract proteins; EMD Biosciences, Inc. La Jolla, CA). The research was conducted with local IRB committee approval, following the tenets of the declaration of Helsinki.

### Enzymatic assays

Aldolase converts fructose-1,6-bisphosphate into dihydroxy acetone phosphate and glyceraldehyde-3-phosphate. Aldolase enzymatic activity assay was measured using the Bayer modification of the hydrazine assay; 3-phosphoglycerate reacts with hydrazine to form hydrazone. Hydrazone absorbs at 240 nm and is quantified spectrophotometrically [5].

Pyruvate kinase catalyzes the conversion of phosphoenol pyruvate plus ADP into pyruvate plus ATP. The reaction kinetics were quantified by coupling with lactate dehydrogenase NADH + pyruvate resulting form lactate + nicotinamide adenine dinucleotide (NAD). Enzyme activity can be measured at 340 nm absorbance by spectrophotometry [6,7].

Isocitrate dehydrogenase catalyzes the conversion of isocitrate into α-ketoglutarate and carbon dioxide. Simultaneously NADP is reduced into NADPH. Characteristic NADPH absorbance quantifies the enzyme activity at 340 nm [6,8]. The assays were standardized with aldolase and pyruvate kinase, L-lactic acid dehydrogenase and glutamic dehydrogenase (A8811, P7768, L1254, and G2501; Sigma Chemical Co., St. Louis, MO).

Malate dehydrogenase catalyzes the interconversion of L-malate and oxaloacetate using NAD as a coenzyme [9]. Malate dehydrogenase activity was determined by measuring decreased absorbance at 340 nm resulting from the oxidation of NADH at 25 °C in 100 mM potassium phosphate buffer pH 7.4 and 6 µM oxaloacetic acid. The malate activity in 10 µg of total tissue extract protein (from glaucomatous and normal TM) was expressed as 10-fold decrease in absorbance and expressed as relative absorbance. Our assay does not distinguish between mitochondrial or cytosolic enzymes and provides a gross measure of malate dehydrogenase activity.

For all activity assays statistical analysis was performed within each group (each group comprised of 10 different tissue samples) and compared to 0.0 using the two tailed one-sample *t*-test.

### Western analyses

Protein was quantified using the biochinchonic acid (BCA) protein assay (Pierce Biotechnology Inc. Rockford, IL). Western blot analysis was performed with 5 µg of protein on 4%–20% Tris-Glycine gels (Invitrogen Inc., Carlsbad, CA). After fractionation, proteins were electro-blotted onto a polyvinylidene fluoride (PVDF) membrane (Millipore, Billerica, MA) using standard procedures and probed with commercially available HNE protein adduct antibody generated in mouse (1:1,000 dilution; NOF Corporation Tokyo, Japan), GAPDH antibody (MAB374; at 1:3 000 dilution); aldolase antibody (AB1809; at 1:1,000 dilution), pyruvate kinase antibody (1:1,000 dilution; AB1235; Chemicon International) and polyclonal antibody to isocitrate dehydrogenase (1:1,000 dilution; PAB-01173; Orbigen Inc.). All secondary antibodies were diluted at 1:1,000.

### Mass spectrometry

To identify the proteins and perform quantification, 80 µg of sample tissue were loaded on to 4%–20% SDS–PAGE gels. Protein bands thus separated were excised, destained with 50% acetonitrile /water, and suspended in 0.5 M triethylammoniumbicarbonate (TEAB; number 17902; Sigma Chemical Co.) pH 8.5 and reduced with 10 mM Tris-(2-Carboxyethyl) phosphine (TCEP; Sigma Chemical Co.). The proteins were subsequently alkylated in the dark using 55 mM solution of iodoacetamide (Catalog No-RPN6302V; GE Healthcare Inc., Buckinghamshire, England) and in-gel digested with sequencing grade modified trypsin (0.1 µg/15 µl in 15 mM N-Ethylmorpholin; catalog number V5113; Promega Corporation, Madison, WI) overnight at 37 °C. The peptides were extracted twice with 50 µl 0.1% triflouroracetic acid/60% acetonitrile and finally with 30 µl of acetonitrile and dried in a SpeedVac. The extracted peptides were incubated with the iTRAQ Rg-8-Plex Assay kit reagents (Applied Biosystems, Foster City, CA). Separate peptides were isolated from different bands and incubated with reagents 113, 114, 115, and 116 (ABI, Foster City, CA) for peptides derived from control TM protein bands, and with reagents 117, 118, 119, and 121 for peptides derived from glaucomatous TM protein bands in 0.5 M TEAB containing 60% v/v isopropanol. The incubation mixtures were dried in a SpeedVac, mixed together and loaded onto a slurry of 500 µl of cation exchange buffer in 12 mM ammonium formate in 25% acetonitrile at pH 2.5–3.0, and separated chromatographically.

### Two-dimensional liquid chromatography (2D-LC) separations

2D-LC is performed to achieve better separation of peptides before mass spectrometry. Peptides recovered after in-gel digestion were labeled with iTRAQ reagents and then subjected to Strong Cation Exchange (SCX) separation.. SCX peptide separations were performed on a passivated Waters 600E HPLC system, using a 4.6×250 mm polysulfethyl aspartamide column (PolyLC, Columbia, MD) at a flow rate of 1 ml/min. Buffer A contained 10 mM ammonium formate, pH 2.7, in 20% Acetonitrile/80% water. Buffer B contained 666 mM ammonium formate, pH 2.7, in 20% acetonitrile/80% water. The gradient was Buffer A at 100% (0–22 min following sample injection), 0% to 40% Buffer B (16–48 min), 40% to 100% Buffer B (48–49 min), then isocratic 100% Buffer B (49–56 min), then at 56 min switched back to 100% Buffer A to re-equilibrate for the next injection. The first 26 ml of eluant (containing all flow-through fractions) was combined into one fraction, then 14 additional 2-ml fractions were collected. SCX fractions were dried down completely to reduce volume and to remove the volatile ammonium formate salts, then resuspended in 9 µl of 2% (v/v) acetonitrile, 0.1% (v/v) trifluoroacetic acid before second dimension separation on reverse phase C18 nanoflow-LC.

For the second dimension separation by reverse phase nanoflow LC, each SCX fraction was autoinjected onto a Chromolith CapRod column (150×0.1 mm; Merck) using a 5 µl injector loop on a Tempo LC MALDI Spotting system (ABI-MDS/Sciex). Buffer C consisted of 2% acetonitrile, 0.1% trifluoroacetic acid, and Buffer D contained 98% acetonitrile, 0.1% trifluoroacetic acid. The elution gradient was 95% C/5% D (2 µl per min flow rate from 0 to 3 min, then 2.5 µl per min from 3 to 8.1 min), 5% D to 38% D (8.1–40 min), 38% D to 80% D (41–44 min), 80% D to 5% D (44–49 min; initial conditions). Flow rate was 2.5 µl/min during the gradient, and an equal flow of MALDI matrix solution was added post-column (7 mg/ml recrystallized CHCA (A-Cyano-Hydroxycinnamic Acid), 2 mg/ml ammonium phosphate, 0.1% trifluoroacetic acid, 80% acetonitrile). The combined eluant was automatically spotted onto a stainless steel MALDI target plate every 6 s (0.6 µl per spot), for a total of 370 spots per original SCX fraction.

### Mass spectrometric quantification

The sample spots mentioned above were dryed and thirteen calibrant spots (ABI 4700 Mix) were added to each plate manually. MALDI target plates (15 per experiment) were analyzed in a data-dependent manner on an ABI 4800 MALDI TOF-TOF. As each plate is entered into the instrument, a plate calibration/ MS Default calibration update is performed, and then the MS/MS default calibration is updated. MS spectra were taken from 5,500 MALDI Spots, averaging 500 laser shots per spot at Laser Power 3100. A total of 3,249 MS/MS spectra were taken with up to 2,500 laser shots per spectrum at Laser Power 3600, with CID gas Air at 1.2 to 1.3×10^−6^ Torr. After the MS and MS/MS spectra from all 15 plates in a sample set were acquired, protein identification and quantitation was performed using the Paragon algorithm as implemented in ProteinPilot 3.0 software (Applied Biosystems/MDS-SCIEX Joint Venture, Foster City, CA) and Matrix Sciences Mascot algorithm version 2.1. ProteinPilot software was used for searches with the following search parameters: Cys Alkylation – Iodoacetamide; ID Focus – Biologic Modifications; Search Effort – Thorough. The Jan 1st 2010 Human NCBI database Sequences containing 512,785 Protein Sequences, plus 156 common laboratory contaminants. For estimation of “False Discovery Rate” (FDR), simultaneous search was performed on a concatenated Decoy database which is the exact reverse of each protein sequence in the database plus 156 common human and laboratory contaminants. Protein identifications at 95% confidence level were retained. The preset “Thorough” (iTRAQ or Identification) search settings were used where identifications must have a ProteinPilot Unused Score >1.3 (>95% confidence interval). In addition, the only protein IDs accepted had a FDR estimation of less than 5%, as calculated from the slope of the accumulated Decoy database hits by the PSPEP (Proteomics System Performance Evaluation Pipeline) program [10]. This FDR estimate is much more stringent than p<0.05 or 95% confidence scores in Mascot, Sequest, ProteinPilot, or the aggregate False Discovery Rate estimations (2× number of Decoy database IDs/Total IDs at any chosen threshold score) commonly used in the literature. Combined with the ProGroup algorithm included in ProteinPilot the FDR gives a very conservative and fully MIAPE-compliant list of proteins identified.

## Results and Discussion

Oxidative stress has been implicated to cause damage to nearly all tissues including trabecular meshwork. Here, we describe that oxidation products may reduce the enzymatic activities of energy metabolism and may become part of the damage process. Thus initial oxidative damage and generated products may have a magnified dampening effect of damage.

**Figure 1:**
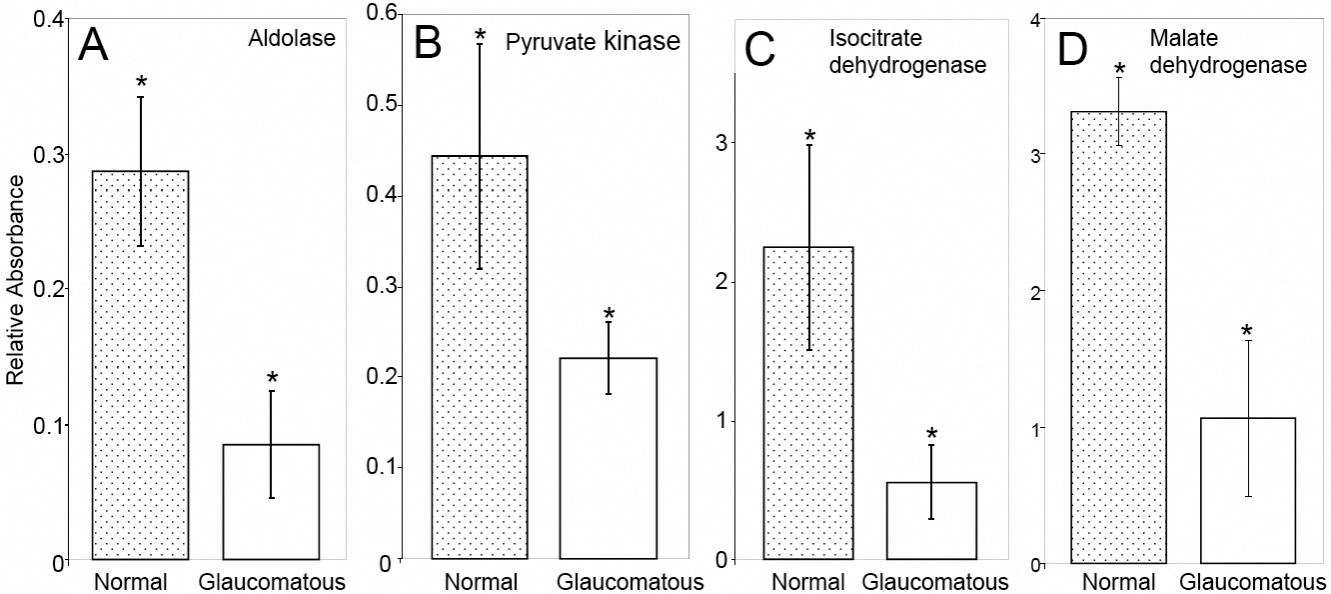
Representative enzymatic activity analyses for aldolase, pyruvate kinase and isocitrate dehydrogenase. Activity measurements were performed on 10 TM tissue samples (5 µg total protein each) derived from normal (dotted bars) or glaucomatous donors (hollow bars). Colorimetric assay was performed for determination of enzymatic activities and standard deviation from 10 individual measurements have been presented. **A**: Activity assay for aldolase, **B**: Activity assay for pyruvate kinase, and **C**: Activity assay measurement for isocitrate dehydrogenase. **D**: Activity assay for malate dehydrogenase. Each bar represents the mean±standard deviation from ten independent experimental readings (ten samples in each group) and was found significantly different from 0.0 for each activity measurement by the one-sample *t*-test. The asterisk indicates a p<0.05.

The activity measurements were performed using standard assay systems for each enzyme for 10 samples per group: normal control and primary open angle glaucoma. All normal TM tissue samples were derived from cadaver eyes from National Disease Research Interchange, Philadelphia, PA. The ophthalmic details for normal eyes followed the tissue delivery. Thus more (about 16 eyes) samples were collected and assayed but only those were included for which details showed definite evidence of ocular health and absence of disease. The trabecular meshwork aldolase, pyruvate kinase, isocitrate dehydrogenase, and malate dehydrogenase activities in the glaucomatous TM tissue were found to be reduced 70, 50, 25, and 69 percent, respectively (Figure 1A-D). Decreased activity is consistent with inactivating modification or degradation of proteins in glaucoma. Microarray studies (GEO accession number GDS359) have been performed by several groups using glaucomatous and normal trabecular meshwork tissue. These microarray studies did not show any discernable difference in the expression level of aldolase and pyruvate kinase at the mRNA level. Our quantitative proteomic analysis also showed decreased levels of aldolase, pyruvate kinase, isocitrate dehydrogenase, and malate dehydrogenase which is consistent with activity measurement (Table 1). We did detect the peptides of the enzymes from excised protein bands that were also immunoreactive for 4-hydroxynonenal (HNE), however, we failed to detect the modified peptides. Our failure to detect the modified peptides may be due to the use of trypsin in the processing of tissues. The failure of detection is explicable based on the possibility that modification might involve lysine and arginine residues which will then not be digested by trypsin. Another possibility is relative low abundance of modified residues. The lipid peroxidation modified proteins have been shown to undergo rapid degradation [2]. Although western analysis will detect the modification due to amplification but it will be difficult to capture the modified peptides by mass spectrometry. Another complication that will also evade capture by mass spectrometry is heterogeneous nature of modification on proteins that will generate a large ensemble of different peptides each with a modification or the same peptide with a modification on different residues.

**Table 1:** Select energy metabolism enzymes down-regulated in glaucomatous TM.

**Accession number**	**Protein**	Peptide matches***	iTRAQ ratio (Glaucomatous/ Normal)*	**Sequence coverage (%)**	SD**
P40925	Malate Dehydrogenase,cytoplasmic	10	0.51	37	0.023
P40926	Malate Dehydrogenase, mitochondrial	10	0.83	40	0.026
P04075	Aldolase	4	0.34	21	0.031
O75874	Isocitrate Dehydrogenase	4	0.62	9	0.007
P14786	Pyruvate kinase,M2 isozyme	4	0.42	9	0.028

The asterisk indicates that the iTRAQ ratio of glaucomatous (labeled with 117–199, 121) with normal (113–116) has been shown. The double asterisk indicates the standard deviation of percentage error from multiple iTRAQ measurement. The triple asterisk indicates that the identified peptides of different proteins against their accession numbers are: P40925: FVEGLPINDFSR; GEFVTTVQQR; ESAFEFLSSA; EVGVYEALK; ENFSCLTR; LSSAMSAAK; SQGAALDK; DDSWLK; DVIATDK; ELTEEK; P40926: VDFPQDQLTALTGR; AGAGSATLSMAYAGAR; GYLGPEQLPDCLK; FVFSLVDAMNGK; GCDVVVIPAGVPR; EGVVECSFVK; IQEAGTEVVK; VSSFEEK; NSPLVSR; KPGMTR; P04075: PYQYPALTPEQK; GILAADESTGSIAK; QLLLTADDR; VLAAVYK; O75874: NILGGTVFR; IIWELIK; SQFEAQK; GLPNVQR; P14786: GDLGIEIPAEK; GSGTAEVELK; VNFAMNVGK; GIFPVLCK.

**Figure 2:**
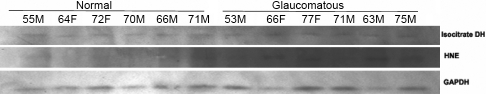
Western analysis for lipid peroxidation product (4-hydroxynonenal; HNE) modification of isocitrate dehydrogenase. Trabecular meshwork protein extract (5 µg) from each eye with donor age and gender as indicated (M, male; F, female) was loaded on each lane and separated over a 4%–20% SDS–PAGE. The proteins were transferred onto a PVDF membrane and probed using polyclonal antibodies to isocitrate dehydrogenase (isocitrate DH). The corresponding protein bands from the region that showed immunoreactive for isocitrate dehydrgenase was excised from an identical gel and electrophoresed and subsequently transferred on a PVDF membrane and probed for isocitrate dehydrogenase (isocitrate DH) against 4-hydroxynonenal (HNE) and against 3-glyceralde dehydrgenase (GAPDH) as indicated.

Lipid oxidation products have been implicated to cause damage to the aqueous drainage system [11]. Our SDS–PAGE analysis suggests the presence of HNE modified isocitrate dehydrogenase protein in the glaucomatous TM tissue compared to controls (Figure 2). Two identical gel electrophoretic separations were performed, protein bands immunoreactive for isocitrate dehydrogenase were excised and subsequently submitted to electrophoresis in a second 4%–20% SDS–PAGE and probed with antibodies to isocitrate dehydrogenase and HNE. The identical gel was probed with antibodies to isocitrate dehydrogenase, HNE and glyceraldehyde-3-dehydrogenase (GAPDH). Western analysis also revealed presence of isocitrate dehydrogenase and other enzymes at relatively high molecular weights indicating aggregate formation as well as locations in gel corresponding to molecular weights lower than the bona fide molecular weights of the proteins consistent with degradation of the proteins (data not shown). However, these analyses necessitated much higher protein loads and longer exposure of detection reagents. While they were thus detected severe darkening of the film overall suggests relative low abundance of aggregated and degraded proteins. Such aggregated and degraded proteins were more frequent and abundant in glaucomatous than normal TM. It is important to note that modification of calpain-1 by isolevuglandin, another lipid peroxidation product was previously observed in the trabecular meshwork, and was associated with both aggregated and degraded calpain-1 [12].

Previously, human growth hormone and its interplay with carbohydrate metabolism have been implicated in open angle glaucoma [13]. Investigations with iodoacetamide, however, were thought not to inhibit energy metabolism [14]. Recently the interest in damage to trabecular meshwork mitochondrial complex proteins and energy metabolism has revived [15,16]. We show here a significant decrease in activities of aldolase, pyruvate kinase, and isocitrate dehydrogenase commensurate with decreased levels of proteins revealed by quantitative proteomic analysis. We used the following iTRAQ reagents (tags 113–116 for normal and 117–119 and 121; ABI Inc.) that showed a lower ratio of peptides in glaucomatous compared to normal TM (Table 1). The sequence coverage of proteins ranged from 9%–40% and the ratio was consistently lower in glaucomatous compared to normal TM (Table1). The ability to capture the aggregated and degraded peptides of these enzymes was likely impaired as detailed above. It is important to note that a low abundance of these enzymes and even lower abundance of their aggregates and degraded products are supported by their relative lack of detection in the western blot compared to calpain-1 or isolevuglandin (iso[4]LGE2) modified products of calpain-1 that we have previously detected in the TM [12]. It is likely that an abundance of aggregated and degraded proteins was low in the TM tissue or that such aggregated and degraded products are cleared faster. Therefore their accumulated levels in the tissue is relatively low. Quantitative proteomics with iTRAQ reagents enabled us to evaluate with a great degree of confidence that the levels of selected metabolic enzymes are lower in glaucomatous TM tissue compared to controls (Table 1). This correlates functionally with lower activity levels for these enzymes (Figure 1). In the future, we will attempt the identify specific sites of HNE modification of these enzymes.

In summary, together with HNE modification of isocitrate dehydrogenase, our results show that energy metabolism enzymes may potentially have reduced activity due to modification or degradation by cellular enzyme cascades in POAG.
